# Pertussis

**Published:** 1914-05

**Authors:** 


**Pertussis.** Inaba, Zeit. fuer Knderheilkunde, has found the Bordet-Gengou bacillus in 78 of 81 cases, in 66 in pure culture. 18 cases, not clinically whooping cough, gave negative results. Mallory & Horner, Jour. of Med. Research, Nov. 1912, have found the same organism and believe that it interferes mechanically with the action of the cilia of the traches and bronchi. They have reproduced the disease and recovered the germ from four young animals. Whooping cough causes 11.4 deaths per 100,000 population, scarlet fever 11.6, measles 12.3, diphtheria only 3.4—indicating relative dangers not ordinarily comprehended. Various authors report favorable results from a vaccine. (Interstate Med. Jour., Jan., 1914).

